# The Future Use of in Vitro Data in Risk Assessment to Set Human Exposure Standards: Challenging Problems and Familiar Solutions

**DOI:** 10.1289/ehp.1001931

**Published:** 2010-06-18

**Authors:** Kenny S. Crump, Chao Chen, Thomas A. Louis

**Affiliations:** 1 Louisiana Tech University, Ruston, Louisiana, USA; 2 National Center for Environmental Assessment, Office of Research and Development, U.S. Environmental Protection Agency, Washington, DC, USA; 3 Johns Hopkins Bloomberg School of Public Health, Baltimore, Maryland, USA

**Keywords:** 21st-century toxicology vision, biologically based dose–response models, in vitro data, point of departure, risk assessment, toxicity pathway models

## Abstract

**Background:**

The vision of a National Research Council (NRC) committee (the Committee on Toxicity Testing and Assessment of Environmental Agents) for future toxicity testing involves the testing of human cells in *in vitro* assays for “toxicity pathways”—normal signaling pathways that when perturbed can lead to adverse effects. Risk assessments would eventually be conducted using mathematical models of toxicity pathways (TP models) to estimate exposures that will not cause biologically significant perturbations in these pathways.

**Objectives:**

In this commentary we present our vision of how risk assessment to support exposure standards will be developed once a suitable suite of *in vitro* assays becomes available.

**Discussion:**

Issues to be faced basing risk assessments on *in vitro* data are more complex than, but conceptually similar to, those faced currently when applying *in vivo* data. Absent some unforeseen technical breakthrough, *in vitro* data will be used in ways similar to current practices that involve applying uncertainty or safety factors to no observed adverse effect levels or benchmark doses. TP models are unlikely to contribute quantitatively to risk assessments for several reasons, including that the statistical variability inherent in such complex models severely limits their usefulness in estimating small changes in response, and that such models will likely continue to involve empirical modeling of dose responses.

**Conclusion:**

The vision of the committee predicts that chemicals will be tested more quickly and cheaply and that animal testing will be reduced or eliminated. Progress toward achieving these goals will be expedited if the issues raised herein are given careful consideration.

The Committee on Toxicity Testing and Assessment of Environmental Agents of the National Research Council (NRC 2007) envisioned a future for toxicity testing that moves from testing in whole animals toward *in vitro* assays conducted in human cells. The vision is anchored to the development of a suite of high-throughput assays for “toxicity pathways,” which are normal biological signaling pathways that when sufficiently perturbed can lead to adverse effects in humans. Developing these assays would be supported by mechanistic computational models that describe the biological circuitry of the pathways. Exposure standards for toxic chemicals would be determined using data from these assays, coupled with physiologically based pharmacokinetic (PBPK) models that predict amounts of chemical reaching cell or protein targets from specific patterns of human exposure, to estimate human exposures that would not cause biologically significant perturbations of these pathways (Andersen and Krewski 2009; Collins et al. 2008; Krewski et al. 2008; NRC 2007).

The committee envisioned that *in vitro* methods will eventually largely or even totally supplant the need for toxicity testing in whole animals. Once in place, these methods are projected to enable toxicity testing to be conducted much more quickly and cheaply than conventional testing in whole animals, which will help to alleviate the large backlog of chemicals that have not been adequately tested. However, implementing this vision will require a large commitment of resources, and the benefits to regulation of toxic substances may be small in the near term. The number of toxicity pathways (TPs) for which assays will be needed has been estimated as high as 200, and the level of effort required to “map” these pathways has been compared with that required to map the human genome. Developing and validating assays for these pathways have been estimated to require $1–2 billion over the next decade or two, and a new national institute with an annual budget on the order of $100 million has been proposed to spearhead this undertaking (Stokstad 2009).

In this commentary, we present our views on how various elements of the NRC vision can be used to develop quantitative risk assessments for toxic chemicals. As plans to bring the NRC vision to life are being developed, the clearer the identification of the direction needed and of the end product, the more likely it is that the eventual product will be maximally useful and developed in a timely and cost-effective manner. The ideas and suggestions in this commentary are offered toward these goals. We focus on issues that can have a major impact on methods for quantitative risk assessment from *in vitro* data but that have not been given sufficient scrutiny up to now. These issues are largely statistical in nature, and we believe will be present in virtually any method for quantitative risk assessment that is based on *in vitro* data.

## Discussions

The NRC vision is predicated upon gaining a thorough understanding of TPs and developing *in vitro* assays that can be used to test for perturbations in TPs. A number of challenging technical problems must be overcome in developing these assays, including developing *in vitro* methods that can be applied to volatile chemicals, addressing effects that take an extended time to develop (including transgenerational effects), accounting for metabolism and addressing cross-cellular and cross-organ feedback signaling (Andersen and Krewski 2009; Conolly 2009; Hattis 2009; Tsuji 2009). Here we make the optimistic assumptions that these problems have been resolved and that *in vitro* assays are available for all important pathways in humans. We focus on issues that must be faced in basing quantitative risk assessments on these assays. Likewise, whereas the NRC vision projects that targeted testing in whole animals will be needed for some time to come, for simplicity the present discussion is limited to use of *in vitro* data.

Although the NRC committee did not define what it meant by an exposure that does not lead to “biologically significant perturbations,” this clearly will be a key issue in implementing the NRC vision. We see four possible definitions: *a*) an exposure that does not result in any biochemical or genomic perturbation of any pathway having adverse sequelae; *b*) an exposure that causes some genomic or biochemical perturbation in such a pathway but does not result in change to any downstream cellular response; *c*) an exposure that causes some genomic or biochemical perturbation that results in a change in some downstream cellular response but that does not increase apical risk; or *d*) an exposure that causes perturbations of these pathways that results in some increase in apical risk, but the amount of increase is not considered to be biologically significant. The last interpretation seems inadequate, because “biologically significant,” however it is defined, is too limited a rubric for determining whether a given increase in risk is acceptable to society. The remaining three possibilities all involve some type of threshold of exposure below which there is no effect upon an apical response, with the threshold occurring at the biochemical or genomic level, the cellular level, or the level of the apical response. So, although not stated explicitly by the NRC committee, its vision appears to tacitly incorporate the notion of an exposure threshold for an apical effect.

Viewed in this way, several issues come to mind. First of all, there is no guarantee that a threshold dose will exist for a particular apical response. Typically, in a large population, there will be a distribution of background responses for an upstream (e.g., biochemical, genomic, or cellular) event in a given pathway, and the tail of this distribution can include values that are associated with adverse apical responses that occur in the absence of exposure to a toxic substance. Consequently, the change in an upstream response necessary to produce an adverse apical response may depend on the baseline response, and it could reasonably be the case that no single amount of perturbation of a biochemical or genomic response will be without risk for all members of a population. A recent NRC committee, the Committee on Improving Risk Analysis Approaches Used by the U.S. EPA, took this view (NRC 2008), concluding that neither cancer nor noncancer effects necessarily have thresholds and recommending risk assessment methods for both cancer and noncancer that assume that there is no threshold of exposure below which no individual is at risk.

Second, the envisioned *in vitro* assays will have limited sensitivity. It will not be possible to confirm that a particular pathway has not been perturbed, but only to set statistical bounds for the potential amount of perturbation. Consequently, every data set is statistically compatible with some level of response at any dose (i.e., no threshold). Because of this limited sensitivity, a threshold dose cannot be proven conclusively to exist (although one’s existence could perhaps reasonably be predicted from a qualitative understanding of underlying mechanisms). Moreover, even if a threshold were known to exist, without making likely unverifiable assumptions about the shape of the dose response, it will not be possible statistically to bound the value of the threshold away from zero.

The NRC vision includes a key role for TP models that would “provide a quantitative, mechanistic understanding of the dose response relationship for the perturbations of the pathways by environmental agents” (NRC 2007). However as we discuss in more detail below, in order to be realistic, TP models will need to be very complex. This complexity will result in large quantitative uncertainties, which make such models particularly ill-suited for quantifying small changes from baseline responses or estimating thresholds.

These issues are conceptually similar to those presently faced in conducting risk assessments using *in vivo* data. The NRC vision is that initially there will be the need for continued reliance on default approaches like those currently applied but that these methods will be replaced by methods that rely on TP models. However, based on the above discussion and the more detailed discussion of the limitations of complex mechanistic models that follows, without some technical breakthrough default methods similar to those presently applied to apical responses will still be needed for the foreseeable future. In the next section we offer some ideas about how these methods might develop. To set the stage for this discussion, we first outline methods presently used with *in vivo* data.

## The Current Paradigm Based on *in Vivo* Data

**Figure 1:**
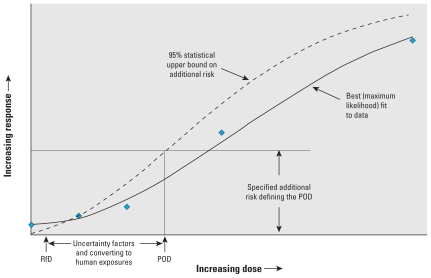
Illustration of an RfD calculation using a benchmark-determined POD.

The method generally used by the U.S. Environmental Protection Agency (EPA) to develop a quantitative risk assessment for a toxic chemical from experimental animal studies involves fitting an empirical dose–response curve to apical responses to estimate a point of departure (POD) defined as an exposure corresponding to a specified adverse change in the apical response (e.g., U.S. EPA 2005). Such a calculation is sometimes referred to as a benchmark analysis, and the resulting POD as a benchmark dose (BMD) (Crump 1984). The change in response used to define the POD is set at a magnitude sufficient to allow it to be measured with reasonable precision in the bioassay (e.g., an increase of 0.1 in the probability of an adverse response). The POD is converted to a human-equivalent POD using quantitative information on the pharmacokinetic differences between the test species and humans, if available, and otherwise using a default method. If the response is cancer and there is no compelling evidence that the dose response is nonlinear at low doses, low-dose risk is estimated by extrapolating linearly toward zero from the point determined by the human POD and the specified increase in response. Otherwise, risk is not quantified, and the human equivalent POD is divided by “uncertainty” or “safety” factors that account for various types of uncertainties (interspecies differences, human heterogeneity, database limitations) to arrive at either a reference dose (RfD) or, for volatile chemicals, a reference air concentration (Figure 1).

Formerly, a NOAEL (no observed adverse effect level) was used in place of a benchmark analysis to define the POD, and it may still be used when data are not available in a form appropriate for a benchmark analysis. Neither a NOAEL nor a BMD can be considered to be risk-free, and at present no factor is applied that explicitly addresses this risk.

## Elements of a Future Paradigm Based on *in Vitro* Data

### Testing strategies

Methods for quantifying gene expression are becoming increasingly sensitive, and some proteins can now be detected in attomolar quantities (e.g., Goluch et al. 2006). However, sensitivity for detecting proteins, or accuracy in measuring proteins, is only one factor of many that can affect the ability to detect the effect of small doses. For example, the accuracy with which cholesterol can be measured does not drive the accuracy of evaluations of intervention strategies in large and diverse populations. To ensure that exposure standards based on *in vitro* data are protective for a population at risk, testing should be conducted in cells from a sample of individuals that is representative of the genetic diversity and disease status of the target population. Cells from sensitive subgroups would need to be appropriately included. To facilitate routine conduct of such testing, libraries of cell lines from many different subgroups (infants, the elderly, asthmatics, diabetics, etc.) could be set up and maintained.

The *in vitro* data needed to set an exposure standard for a substance are expected to consist of responses from a suite of *in vitro* tests for the identified TPs, measured in cells from many different individuals at a number of different exposure concentrations. For example, if the suite of tests for TPs includes 20–100 tests, and each test is applied to cells from 1,000 individuals at 15 different concentrations, the data set would consist of 300,000–1,500,000 data points.

### Calculation of a POD

**Figure 2:**
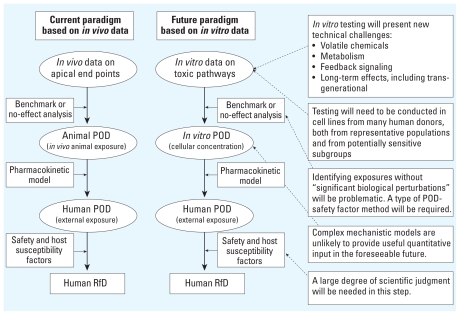
Comparison of current and envisioned risk assessment paradigms.

We envision that for the foreseeable future any risk assessments to support exposure standards developed from *in vitro* data will need to use methods conceptually very similar to the approaches described above that are currently used with *in vivo* data (Figure 2). *In vitro* data derived from human cells will be used to calculate a POD defined as a cellular concentration. A PBPK model will be applied to convert an *in vitro* POD to a human POD. The human POD will be divided by various factors to produce a human RfD, although these factors will need to differ both in rationale and in numerical value from those presently applied to *in vivo* data.

The POD could be based on either a benchmark calculation or some type of no observed response dose similar to a NOAEL. Whereas the benchmark approach avoids some of the conceptual problems associated with a no observed response approach (e.g., Crump 1984), its use requires selecting a dose–response model and positing a level of increased risk—decisions not needed in calculating a no observed response dose. Moreover, with the greater flexibility of *in vitro* methods in selecting exposure levels to evaluate and in controlling the sensitivity of the tests, some of the advantages offered by a benchmark approach may be less important. The NRC (2007) applied a benchmark approach in its illustrative examples. A NOTEL (no observed transcriptional effect level) has been proposed for use with gene transcription data (Ankley et al. 2006).

With either a benchmark or a no-observed-response approach, decisions will be required regarding what *in vitro* responses to account for in their calculation. Should they be protective against any change, or should some biochemical changes be considered to be adaptive and nonadverse? Also, it may not be straightforward to extract a POD from the large amount of data projected to be generated. If a large number of pathways require testing, false positives could be a serious problem. Decision rules will be needed to deal with these issues.

*In vitro* testing has an advantage over what is typically the case with *in vivo* testing in that replicate cells from each individual can be tested at each concentration, so that each individual can serve as its own control. This permits more realistic models to be used in POD calculations, such as ones that allow for the amount of change in response from background to depend upon an individual’s background response as well as exposure.

We do not foresee a useful role for quantitative estimates of low-dose risk obtained by extrapolating downward from the POD. An extrapolated risk of a change in biochemical, genomic, or cellular end point would be much more difficult to interpret than the risk of an adverse apical end point. Although conceptually a change in a biochemical end point could be translated into an increase in apical risk using biologically based dose–response (BBDR) models of downstream processes, as explained in more detail in a following section, developing reliable models for this purpose will be extremely difficult.

### Uncertainty factors

We suggest that one factor should be applied to the POD to account for the severity of the downstream apical end point. This would allow responses in pathways leading to life-threatening diseases to be regulated more stringently than those leading to less serious apical end points (e.g., cancer vs. a 5% change in liver weight).

Uncertainty factors should also reflect what is known about the quantitative relation between the *in vitro* end point and the apical adverse effect. For example, suppose the POD is defined as the *in vitro* concentration corresponding to a 10% increase in Nfr2 pathway activation. Ideally, some quantitative notion of the downstream consequences of a 10% increase in this pathway should be incorporated into the selection of the uncertainty factors. At this point we do not know if such relationships can be reliably quantified. It seems likely that uncertainty factors will need to be developed by consensus based on a qualitative understanding of the mode of action of the toxic substance and the potential shape of the dose response for this mode of action. Although it has been proposed that BBDR models can eventually be used to quantitatively link biochemical and cellular responses to apical effects (Conolly 2009; Rhomberg 2009), as discussed in the next section, it appears that it will not be possible to develop such models from TP testing data in the foreseeable future.

The POD may be calculated from the “most sensitive end point,” which possibly could be the *in vitro* response for which a statistically clear perturbation is seen at the lowest concentration. However, different end points may differ in their effect upon the apical response even though they have similar dose responses. If there were sufficient understanding of the downstream effects of different end points, such differences in sensitivity could be accounted for by defining different uncertainty factors for different *in vitro* end points.

In the current risk assessment paradigm based on results in whole animals, factors such as polymorphisms, and preexisting diseases are generally treated empirically through the application of uncertainty factors. However, with *in vitro* data it may be possible eventually to account for such factors directly through the selection of the population of cells for testing. For example, unique risks to persons with preexisting disease or certain polymorphisms could be accounted for by including cells from such persons in the *in vitro* assays.

### Role of mechanistic models

Two types of mechanistic models have potential application in the NRC vision. BBDR models describe biological processes at the cellular and molecular level to link external exposure to an adverse apical response. TP models link cellular concentrations of toxicants to predictions of perturbations of TPs at the cellular level but do not model apical responses (NRC 2007). Both types of models will typically involve complex mathematical formulations and require estimates of a number of biological parameters as inputs.

Experience to date with complex models developed from *in vivo* data can assist in evaluating the potential uses and limitations of such models derived from *in vitro* data. Whereas BBDR models based on *in vivo* data have proved useful in elucidating mechanisms of action of toxic chemicals and identifying knowledge gaps, they have not proved to be useful in estimating low-dose risks in the range of risks required for setting exposure standards (e.g., 10^−3^ and lower). There are several reasons for this. In order to link exposure with risk of an adverse apical response in a BBDR model, at least one of the intermediate variables in the model (e.g., a cellular response) must be a function of external exposure, generally through a PBPK model. The dose response of such an intermediate variable is usually modeled empirically, making the resulting estimated risk of the apical response subject to the same uncertainties as direct empirical modeling of the apical response itself.

This problem is exacerbated by other problems inherent in complex models, including variation introduced by inability to measure different intermediate responses in the same individuals, uncertainty in the relevance of measurements to the mechanism in question, and uncertainty as to whether the mechanism being modeled is the correct one (Crump et al. 2010). These problems are particularly acute if the toxicant affects multiple and possibly mutually interacting intermediate steps in the disease process. These complexities manifest as uncertainties in estimated risk that often are much larger than the small risks that the model is attempting to estimate.

Such problems will also be present in any BBDR model developed from *in vitro* data. Moreover, they will be exacerbated by inherent limitations in using *in vitro* data to quantitatively predict *in vivo* responses. The NRC committee did not include a role for such models in its vision, stating that “the committee sees BBDR-model development for apical end points as part of a much longer range research program and does not see routine development of the models from TP testing data in the foreseeable future” (NRC 2007). However, others (e.g., Conolly 2009) consider development of such models from *in vitro* data to be both feasible and important for protecting of public health while avoiding unnecessary economic consequences. Although we agree conceptually that reliable models would be very useful for these applications, we also agree with the NRC committee that it is highly unlikely that it will be possible to develop such models in the foreseeable future.

In contrast to BBDR models, the NRC vision included a central role for TP models that quantitatively model the cellular responses in terms of more basic biochemical and genomic responses. In the schematic in Figure 2, TP models would replace both the POD and some of the uncertainty factor components. Although the examples provided by the NRC committee used a POD uncertainty factor approach, this approach was projected to be used only in the near term while the requisite TP models are being developed.

We envision that TP models will prove useful in gaining understanding of TPs and in designing assays for them. We are less optimistic regarding the ability of TP models to contribute quantitatively to risk assessments that support exposure standards. As detailed in the discussion that follows, we have two reasons for this: First, predictions of small changes in responses from complex models are inherently uncertain; second, many of the responses that such models would be used to predict can be measured directly in *in vitro* assays.

Most of the problems described above that complicate efforts to use BBDR to estimate low-dose risk will be present in TP models. TP models must be quite complex to realistically reflect the complexity of TPs. For example, a model cited by the NRC committee for how *Escherichia coli* protect against heat shock (El-Samad et al. 2005) consists of a set of 31 differential-algebraic equations with 27 kinetic parameters, data for many of which are not yet available. Even if such a complex TP model is correctly specified, uncertainty in parameters can have a very high leverage on estimates of low-dose response, so what one gains from introducing more biological realism through the modeling will likely be lost in a sea of statistical uncertainty (Crump et al. 2010). However, this does not mean that a TP model is useless for risk assessment. A TP model could be used to evaluate theoretically various stages in a TP that are exposure dependent and important to the apical response and thus play an important role in helping to identify critical *in vitro* end points that can serve as biomarkers for apical end points.

For TP models to be used quantitatively in risk assessment, they must be dose–response models; that is, they must model the downstream cellular response, not just in terms of upstream genomic or biochemical responses, but mechanistically as a function of the cellular concentration of the toxic substance or its metabolites. Whether such modeling will be possible at all and, if so, whether it can be implemented with sufficient accuracy are open questions. For example, the heat shock model, even though already quite complex, does not include modeling of the dose response resulting from the perturbation of this system by a toxicant. The same is true of the other TP models cited as under development by the NRC committee. Without this critical component, genomic responses to toxic insults will need to be modeled empirically and will be subject to the limitations inherent in such modeling.

These limitations will be magnified if, as will likely be the case, a number of genomic or biochemical responses must be modeled, or the dose response of the cellular variable being modeled depends in a complicated way on the dose responses for the biochemical or genomic responses, for example, if the cellular response is very sensitive to small differences in these responses. Such models will also be subject to limitations stemming from difficulties of *in vitro* systems in capturing the complexity of responses in whole animals, for example, complexity resulting from intercellular signaling pathways that are present *in vivo* but are not present, or are present in a modified form, *in vitro*.

The cellular responses that TP models would be designed to predict may be amenable to direct measurement. In fact, having such data may be necessary to fully implement a TP model, just as data on the apical response have been used in the development of BBDR models for *in vivo* responses, (e.g., Conolly et al. 2003). Given the problems inherent in complex models that we have discussed, and the relative ease with which *in vitro* data can be generated, we foresee measurements of critical *in vitro* end points playing a more important role than predictions from TP models in risk assessments based on *in vitro* data.

Rather than basing a risk assessment on predictions of a cellular response made from a TP model, an assessment could be based more directly on upstream responses that would otherwise serve as inputs to a TP model. In other words, once the dose-related biochemical and genomic steps in a pathway have been identified, it may be more feasible to work directly from these inputs rather than from predictions of downstream consequences obtained from TP models.

Once developed, a TP model would quantitatively predict some downstream cellular response, such as apoptosis, as a function of gene expression or other biochemical end points. As is true with any such model, such predictions will be subject to statistical uncertainty. Consequently, just as the NRC committee proposed benchmark methods for dealing with statistical uncertainty in measured responses, similar methods would be needed to deal with uncertainty in predictions from TP models. Thus, even if successfully developed and employed, TP models would not obviate the need for POD-type methods.

## Conclusions

We have described our vision of how *in vitro* data will be used in the future in the conduct of quantitative risk assessments for toxic chemicals. We foresee that these data will be used in ways that are conceptually similar to the POD uncertainty factor approach presently used with *in vivo* data: *In vitro* data will be used to determine PODs using either BMD- or NOAEL-type approaches, to which uncertainty factors will be applied. Calculation of PODs will be much more complicated than similar calculations using *in vivo* data because of the multiplicity of TPs and large amount of data that are expected to be available on each pathway. Determining suitable uncertainty factors to apply to these PODs will require larger inputs of scientific judgment than those based upon *in vitro* data because of the uncertainty in the quantitative relationship between the cellular response upon which the POD will be based and a downstream apical response.

For these methods to be used by regulatory agencies, numerous decision rules will be needed to guide methods for calculating PODs and selecting uncertainty factors. In the past, incorporating such approaches into regulatory decisions has been a very time-consuming and contentious process. Even though we are in the very early stages of developing a risk assessment paradigm based on *in vitro* data, it is not too early to begin thinking of what changes in environmental laws and institutional arrangements will be needed to facilitate this process (Elliott 2009).

We recognize that the uncertainty-factor–based approach is not the most comprehensive approach and that selecting appropriate uncertainty factors will be a difficult and likely confrontational process. However, these problems are also inherent in current methods. There is a critical need to move forward in using the new data as soon as possible. Even if more comprehensive methods eventually emerge, progress in the foreseeable future is most likely to occur using simpler methods like those outlined in this commentary.

One facet of the NRC vision that we believe has been given insufficient scrutiny is the role of quantitative TP models in risk assessment. The vision assumes that, once such models are developed, they will replace POD–uncertainty factor approaches. However, details of how this will come about have not been clearly articulated. Even if this optimistic goal were to be realized, it would not negate the need for POD–uncertainty factor approaches, because every model is limited in its predictive ability. However, we think the goal is unlikely to be realized in the foreseeable future. Such models typically model downstream responses as a function of more basic biochemical and genomic end points but do not incorporate the critically needed modeling of the dose responses of these end points. In addition, complex models of this type, even if correctly specified, are fraught with statistical uncertainty, which severely limits their usefulness in making estimates of small changes in response that are needed in risk assessments to support exposure standards. Because of these difficulties, and also because of relative ease of collecting *in vitro* data, we expect that measurements of critical *in vitro* responses will prove more useful than will predictions from complex models.

We recognize that we may be overly pessimistic in our assessment of the utility of TP models, and we encourage continued research in this area. At the same time, we also urge more careful consideration of the role of TP models in quantitative risk assessment and the likelihood of their success in fulfilling this role.

Use of *in vitro* data in risk assessment has great promise toward allowing chemicals to be tested more quickly and cheaply and for reducing or eliminating the need for subjecting animals to toxic insults. It is our hope that the bar for accepting approaches based on *in vitro* data will not be set too high. In view of the numerous serious limitations of current approaches, results from these methods based on whole-animal data should not be held up as gold standards. This point is particularly important considering that almost all whole-animal data are obtained from high doses that may operate through different sets of TPs than do low doses.

The discussions presented in the article are offered to help facilitate the kinds of thinking and discussion that will be required to bring these modern *in vitro* methods into general use for setting exposure standards for chemicals. Large commitments of resources will be required to develop and validate the *in vitro* tests that will be needed. These resources can be allocated most efficiently if there is a clear vision of how these tests will be applied in setting exposure standards. There is, of course, the possibility of a technical breakthrough that will render obsolete the methods we have outlined here. Even if such a breakthrough does eventually occur, we believe that methods like those we have discussed here will be required for some time.
